# Observations on the Surgical Anatomy of the Head and Neck : Illustrated by Cases and Engravings

**Published:** 1816-01

**Authors:** 


*Observations on the Surgical Anatomy of the Head and Neck: Illustrated by Cases and Engravings.*

By ALLAN BURNS, Member of the Royal College of Surgeons, London; and Lecturer on Anatomy and Surgery, Glasgow. 8vo. pp. 415. Edinburgh, 1811.

THE subject which the book here announced is destined to illustrate, and the character and circumstances of the man by whom it was written, conspire to give it a deep and peculiar interest. No region of the human body presents, with respect to anatomico-chirurgical research, a wider or more important field than those so ably delineated in this work. It is the last production of Allan Burns, and unquestionably his best. As such, we never open it without mingled feelings of sorrow and veneration. Perhaps an opportunity may ere long be afforded us of paying to the memory of that estimable young man the public eulogium due to his talents and exertions. In the mean time, the very fact of presenting, at a period so remote from its publication, an analysis of the work before ns, will express, with more than the eloquence of language, our sense of his character and endowments. Impelled to this deviation from the common path of criticism, by the hope of public utility, we deem such incentive our best apology—indeed the only one that can be required of us. Frequently have we observed with regret, that the contents of this interesting volume are little known or inadequately appreciated among the great mass of medical students and practitioners; and rich will be the compensation of our labours, if they shall contribute to diffuse a knowledge of its real value, and thus to acquire universally for the name of Allan Burns, that exalted rank which it has already attained in the estimation of all the more enlightened and philosophic members of the medical profession in these islands.

To accommodate the method of investigation to the precise nature and circumstances of any object or production respecting which we are solicitous to obtain correct ideas, constitutes, in criticism as in every other branch of science, an art highly conducive, if not essential, to ultimate success. Thus, in examining the present publication, which comprehends, intimately blended with each other, the structure and pathology of the facial and cervical regions, we shall forsake our wonted route of regular analysis, and exhibit the two divisions in an isolated form, and under two distinct articles. The first of these will be occupied by anatomical description; the second, by surgical remarks and operations. Upon this plan, the anatomical or surgical inquirer will find the object of his research unconnected with other matter, and delineated with a clearness and precision unattainable in the state of combination; and the great inconvenience experienced in analyzing a work remarkably defective in arrangement,* will be somewhat obviated. The present then will form a strictly anatomical article.

In the public schools of medicine, British as well as continental, the study of surgical anatomy is, in general, much too little cultivated. Such neglect is inexplicable, when we consider that this is the very object upon which all the researches of the young surgeon, all his labours in tracing the structure of the human body, should be mainly directed. It is a knowledge which nothing but long, and patient, and reiterated dissections can teach; a knowledge, the value of which is seldom correctly estimated until the opportunities of acquiring it are irrevocably past. Only amid the disastrous scenes of a field of battle, or in the more trying exigencies of domestic practice, where the character and fortune of the surgeon are at stake, and where the self-possession alone, resulting from conscious ability, can rescue him from embarrassment and disgrace, does his mind become duly impressed with the insufficiency of the common mode of anatomical instruction. Then only is it that the views and principles, which should have regulated his studies in the dissecting-room, begin to be developed; and then, too frequently, does he turn with an eye of unavailing anguish and regret to that period when the field of knowledge lay open before him, and a right impulse and direction were only wanting to insure to his labours a rich and imperishable harvest. How many a young surgeon is there, who, fluently as he may describe the structure and distributions of the nervous system, would feel terribly confounded, if called upon to expose the infra-orbitary branch of the fifth pair, or cut through the great facial trunk in its passage from the stylo-mastoid orifice. We have known a man, who could trace, in eloquent language, all the ramifications of the sanguiferous tubes, cut across the belly of the Sartorius muscle in search for the femoral artery, and, after all, abandon the opera-

* The work is almost without chapter or division.

tion in disgrace; and, in our younger days, he who, without division of a muscular fibre, could expose the anterior tibial vessels, was esteemed as singularly expert. These things should not be; and would not, if teachers of anatomy, in their lessons and writings, instead of exhibiting each organ and system under a detached and isolated view, would combine their descriptions, and trace accurately the relation which each bone and muscle, vessel, nerve, and viscus, bears to others in all the varying circumstances and situations of the human body. Greatly then are we indebted to Mr. Allan Burns for this attempt to elucidate the surgical anatomy of a region, not more intricate in structure than interesting from the accidents and diseases to which it is obnoxious: and deeply do we deplore, that he did not live to extend his researches, with equal spirit, and accuracy, and success, to other regions quite as important, and hitherto very inadequately delineated by British writers on anatomy.

Of the objects and nature of the work, the introductory observations of Mr. Burns will convey an accurate notion to our readers.

"In works on anatomy, each separate system is generally considered apart, and without a reference to the others; and, in surgical books, it is presumed that the student has already acquired a sufficient knowledge of the structure of the human body. Although we have, perhaps, little reason to complain of the want of tolerably accurate descriptions of the bones, the muscles, the blood-vessels, the nerves, the absorbent system, and the glands; still, this to the operator is not sufficient. It requires a greater degree of discrimination, and a more accurate conception of the parts, than most students, nay, I believe, than most surgeons possess, to be able to combine these disjointed lessons, so as to form from them a useful and connected whole. I am afraid that, in planning operations, the surgeon, too frequently, proceeds on a limited view of the parts amongst which he has to cut. More than once, I have heard the propriety of an operation argued from the inspection of a mere blood-vessel, and dried preparation; a guide surely more liable to mislead, than to lead to a rational practice. The blood-vessels are, no doubt, highly necessary to be perfectly understood; but this knowledge, to be practically useful, must be conjoined with a comprehensive acquaintance with the neighbouring parts. On this account, I endeavour to connect the demonstrations of the arteries with the local structure of the muscles, nerves, and glands, and with the performance of surgical operations. That this is the most advisable plan of teaching the student the true value of anatomy few will dispute; but I fear that the execution will not prove equal to the design." Page 1—2.

We now proceed to consider *the surgical anatomy of the neck.* The platisma myoides muscle, lying directly beneath the integuments, and attached to the cellular membrane of the jaw-bone, covers the neck anteriorly and laterally. It contributes, by its action on the angle of the mouth and cervical integuments, to express the passions of rage and grief; but the support of the deep-seated parts constitutes its principal office.

*The cervical fascia* is a membrane of thin but resisting texture, descending from the base of the lower jaw; thinner at the front than at the angle of the jaw, where a fold of it dips in and adheres to the styloid process; and, uniting with an aponeurosis from the pterygoid muscle, forms the ligament of the jaw.* In its course along the neck, it dips down among the muscles and glands, enveloping the latter in these capsular productions from its inner surface: external surface smooth. It is uniform in thickness below the hyoid bone; but assumes, in expanding over the pectoral muscle, a cellular appearance: it covers, at the inferior part of the throat, the sterno-mastoid muscles, and stretches between their tendons. Here also it is double; and a large mass of fat, often inclosing a conglobate gland, lies imbedded between the layers; the more deep-seated of which, tense, fibrous, and much stronger than the superficial, covers the outer surface of the sterno-hyoid and sterno-thyroid muscles; preventing the passage of the finger from the surface into the chest, or from that cavity outward beyond the lower margin of the thyroid gland. Interlacings of tendinous fibres strengthen the fold from the superficial layer, where inflected along the superior edge of the sternum. Mr. Burns first discovered this fascia about the year 1804, in an anasarcous subject. On the emaciated and dropsical, it will be most advantageously examined. The influence which it exerts in modifying the morbid affections of the cervical region, will be explained in our concluding article. †

In tracing the anatomy of *the lateral and lower part of*

---

* This ligament extends, chord-like, from the angle of the jaw backward and downward, and becomes more distinct on bending back the head, and inclining it to the opposite side. In emaciated subjects, it may be seen losing itself about the anterior margin of the sterno-mastoid muscle.

† Mr. Burns describes here the situation of the thymus gland; but he should have remembered, that it could not be correctly introduced in a delineation which applies almost exclusively to *adult* age. REVIEWER.

*the neck,* the subclavian arteries first present themselves, mounting upward and outward from their origin; covered, until reaching the *scaleni,* by the sterno-mastoid muscles; connected, between the scaleni and aorta, with the eighth recurrent laryngeal, sympathetic, and phrenic nerves; subclavian vein; and intimately, on the left side, with the termination of the thoracic duct. By raising the sternal attachment of the sterno-mastoid muscle, the eighth nerve is seen lying on the fore-part of the subclavian artery, behind the sternal extremity of the clavicle, and just opposite the eighth nerve, behind the artery, the lower cervical ganglion of the sympathetic. The right recurrent nerve hooks round the subclavian artery; climbing, in its way to the larynx, along the tracheal side of the sympathetic: the left turns round the aortic arch, and ascends between the subclavian artery and œsophagus. Anteriorly to the artery lies the subclavian vein; alternately sinking lower towards the chest, and swelling over the artery during life, according as it is flaccid or tense: The former is its state in the dead subject. The thoracic duct enters it about the eighth of an inch nearer the acromion than the point at which it is joined by the internal jugular vein. The termination of the duct corresponds to the space between the sternal and clavicular portions of the sterno-mastoid muscle.

If the occiput be turned back, and an incision drawn from the angle of the jaw to the coracoid process of the scapula; a second, from half an inch behind the mastoid process to the acromial edge of the origin (clavicular attachment) of the sterno-mastoid muscle; and a third, along the superior margin of the clavicle, a triangular space will be described. This, on removing the integuments and fascia, will be seen divided into two unequal portions by the posterior belly of the omo-hyoideus muscle. In the superior portion are lodged small conglobate glands, nervous branches, art-transversalis colli, and often the cervicalis superficialis: in the inferior, the subclavian artery, accompanied by its plexus of nerves above and behind. Here the artery must be exposed in the operation of ligature. The course of the omo-hyoid muscle in the living subject may be traced by a line drawn from the junction of the coracoid process with the clavicle, to the sterno-mastoid muscle, two inches, in the adult, above the sternum.

The situation of the deep-seated parts about the neck and face is next to be described; and, for this purpose, the adult subject is selected, as offering a specimen of the most perfect formation. The variations, dependent on age and other circumstances, are afterwards to be investigated.

In the living subject, at perfect quietude, when the basis cranii is placed parallel to the horizon, the hyoid bone presents beneath the integuments four fingers' breadth behind the chin, and a quarter of an inch lower than the base of the jaw. About half an inch below the base of the hyoid bone, the superior margin of the thyroid cartilage projects, and gradually declines from the perpendicular as it goes backward: between these bodies, there is anteriorly a small hollow; below the thyroid cartilage, another, still less. About four lines below this cartilage, the cricoid shews its prominent semicircle. Beneath this latter, and between the margins of the sterno-mastoid muscles, the obscurely defined substance of the thyroid gland is felt. An angular hollow, situated still lower towards the sternum, contains the trachea. Six fingers' breadth is commonly found between the superior margin of the thyroid cartilage and sternum. At mid-distance between, is the superior margin of the thyroid gland. One finger's breadth being allowed for the gland itself, two fingers' breadth remain between the base of the gland and the sternum. If, however, the occiput be completely thrown back, as is commonly done in operations about the throat, the distance between the chin and sternum is increased to twelve fingers' breadth; and the thyroid cartilage, instead of lying, as in the natural position, more than three fingers' breadth behind the chin, is but one thumb's breadth below a line extended from the chin to the sternum. In this posture, a space of nearly five fingers' breadth intervenes between the chin and superior margin of the thyroid cartilage; and somewhat more than three fingers' breadth between the superior margins of the thyroid cartilage and gland. Then, deducting the breadth of the gland itself, *three* fingers' breadth, instead of *two* found in the natural situation of the parts, remain between the gland and sternum. Thus, by bending back the head, one fingers' breadth is gained in performing the operation of tracheotomy.*

* This is an important observation, which cannot be too deeply impressed on the surgeon's mind, in operating upon the trachea By making the incision in tracheotomy too low down, the great subclavian vein may be wounded where it crosses at the superior margin of the sternum. We have absolutely seen this fatal blunder committed.

*Reviewer.*

On removing the integuments, external jugular vein, platysma myoides, and fascia, the sterno-mastoid muscle comes into view, crossed posteriorly by the omo-hyoid, opposite the upper margin of the cricoid cartilage, and four fingers' breadth above the clavicle. A more certain mode of ascertaining precisely the point where these muscles cross, consists in extending one thread from the anterior part of the mastoid process to the centre of the upper bone of the sternum, and another from the side of the body of the hyoid bone to the central part of the clavicle. The common carotic trunk, usually lies beneath the point of intersection of these two lines. Above this point, the course of the artery may be traced by a thread extended from thence to (the angle of) the jaw-bone.

The common carotid, from the root of the neck to its division, is surrounded by important vessels and nerves. The eighth nerve and internal jugular vein are inclosed in the same sheath with it. The sympathetic nerve lies between the sheath and longus colli muscle. The jugular vein is on the acromial side of the artery ; the eighth nerve, between them (posteriorly). The ramus descendens noni runs on the anterior part of the carotid artery to be distributed to the omo-hyoid, sterno-hyoid, and sterno-thyroid muscles. This nerve, above the point of decussation of the sterno-mastoid and omo-hyoid muscles, receives contributions from the second, or second and third, cervical nerves. These twigs pass to the descendens noni, between the common carotid artery and internal jugular vein. A small swelling is often seen on the nerve where they join it; and from hence, the neighbouring muscles are supplied. The descendens noni may be placed either in, or externally to, the carotid sheath : in the latter case, the cervical twigs cross to it over the anterior surface of the internal jugular vein.

For the purpose of tracing the situation and depth of the nerves and blood-vessels in the neck with greater precision, three ideal regions may be distinguished. *The middle region,* of nearly triangular shape, will be described by three lines : the first drawn from the root of the mastoid process to the junction of the horn and body of the hyoid bone; the second, from the anterior edge of the mastoid process to the centre of the clavicle. Along the whole of this region, the carotid artery with its accompanying veins and nerves, is covered only by the common integuments, platysma myoides, cervical fascia, and investing sheath.

Lower down in the neck, the artery is more deeply seated,* and covered, in addition, by the sterno-mastoid, sterno-thyroid, and omo-hyoid muscles. Higher, it is concealed behind the angle of the jaw.

In this middle region are situated other important parts, besides those already mentioned. Opposite to the division of the common carotid, a slender branch, *nervus superficialis cordis,* is sent off by the superior cervical ganglion of the sympathetic, descends along the tracheal margin of the carotid artery, is increased by numerous twigs from all the neighbouring nerves; and, after interweaving with those of the recurrent at the root of the neck, gets attached to the aorta; and thus is conducted to the heart. Glandulæ concatenatæ also accompany the carotid artery; some situated anteriorly; others, between the vessel and the spine. A few lines above the upper margin of the thyroid cartilage, the superior laryngeal nerve emerges from behind the internal carotid, and directly enters, with the superior laryngeal artery, behind the hyo-thyroid muscle. The thyroid branch of the superior thyroid artery is accompanied by a small twig from the eighth nerve. Between the os hyoides and thyroid cartilage, a small gland, not larger than a millet-seed, lies imbedded in fat: it is immediately beneath the hyo-thyroid muscle; and only separated from the bag of the pharynx and epiglottis by the membrane connecting the bone and cartilage. In this middle region, the common carotid, superficially situated, usually divides into external and internal. The exact point of division varies. It is commonly opposite the superior margin of the thyroid cartilage.† Of the two divisions, the external carotid lies most superficial, and nearer to the pharynx; hence somewhat remote from the nerves and jugular vein, with which the common and internal carotids are actually in contact.

The intimate structure of the thyroid gland requires no

---

* "Mr. John Bell asserts, that the carotid is deeper the farther it retracts from the chest. Mr. Bell's description is only applicable to a front view of the neck; in which case, as the larynx projects, the artery *seems* to be thrown back." Page 72.

† Many variations, which it would be needless to specify minutely, occur respecting the mode, and point, of division of this artery. Sometimes it divides very low in the neck, but rarely so high as the angle of the jaw: consequently, Mr. John Bell's description is very incorrect. Surgeons, however, will do well to bear in mind, that great varieties exist with respect to the course and arrangement of these vessels.

extended description: it consists internally of cells not freely communicating with each other. Its situation on the front of the trachea has already been traced. The two lateral lobes, composing it, are united by a slip* which crosses the trachea a few lines below the thyroid cartilage. The upper margin of the gland is parallel to the second ring of the trachea. It is covered anteriorly, but not completely, by the sterno-hyoid muscles; laterally by the sterno-thyroid and omo-hyoid. Above these muscles, the upper peak of the lateral lobe, embracing the cricoid cartilage, is seen.

*In the lower cervical region,* the left carotid artery lies on the outer edge of the œsophagus; which projects from beneath the trachea, is covered by twigs of the recurrent nerve, and traversed by the inferior thyroid artery in its passage to the gland. The left lobe of the gland covers the commencement of the œsophagus; and is supported in this situation by the sterno-thyroid muscle. Between the muscle and gullet, a cluster of conglobate glands is found. The pharynx terminates in the œsophagus after it has passed the lower border of the cricoid cartilage. Its contraction is very gradual.

*Description of the parts situated above the digastric muscle; and anatomy of the angle of the jaw.*—The position of the skull greatly influences the relative situation of these parts: hence some circumstances in its mechanism must first be considered. From the manner in which the cranium is attached to the spine, a considerable space is left between the anterior surface of the vertebral column and vertebral surface of the lower jaw. If the basis cranii be parallel to the horizon, the surface of the teeth, in the upper jaw, corresponds nearly to the line of the great occipital hole. The transverse diameter of the meatus auditorius externus is only interposed between the mastoid process of the temporal bone and ramus of the lower law. The latter, in the well-formed adult, is about two inches long; and the angle situated about one inch anteriorly to the cervical

---

* Mr. Burns, in one instance, has seen this slip passing *between* the trachea and œsophagus. Such a peculiarity of structure, he observes, is much to be dreaded; since, in the event of a morbid thickening of the slip, incurable difficulty of breathing and deglutition would ensue. With respect to the physiology of the thyroid gland, Fodéré, in his *Essai sur le goitre et cretinisme,* asserts, that its excretory ducts have been traced, by minute injection, opening on the surface of the tracheal membrane.
REVIEWER.

portion of the spine. Just before the root of the mastoid process, but nearer towards the centre of the cranial base, the styloid process descends in an anterior direction; so that its distal extremity is concealed behind the ramus of the lower jaw. This character is peculiar to adult age.

In the horizontal position of the cranium, if the throat be at rest, the pharynx lies flat; and on its posterior surface, which is directly in contact with the face of the vertebral column, the back part of the larynx rests. The hyoid bone is then almost as high as the base of the lower jaw: and, while the posterior belly of the digastric muscle declines but slightly forward, the anterior portion takes an almost horizontal direction to the jaw-bone. Beginning the description from behind forward, we see the spinal accessory nerve between the transverse process of the atlas and internal jugular vein; an incision opposite this process, along the anterior border of the sterno-mastoid muscle, will expose it: lower down, it is covered by that muscle, which it perforates in passing to the trapezius. In contact with this nerve, nearer the angle of the jaw, is the internal jugular vein: next, the lingual nerve; and internal carotid artery, deep seated, and separated from the external, by the styloid process, or by the ligament, if that process be short, which stretches from it to the appendix of the hyoid bone. The occipital artery arising from the external carotid just below the angle of the jaw, slants upward and outward, traverses the internal carotid, eighth and lingual nerves, and internal jugular vein; passes behind the digastric muscle, round the mastoid process, and above the transverse of the atlas. A little below the line of the lower jaw, the lingual nerve comes from between the internal carotid and jugular vein; and turning abruptly forward, often hooks round the root of the occipital artery: Here it gives off the ramus descendens noni; then passing behind the termination of the facial vein, and in front of the external carotid, dips, nearer to the hyoid bone, behind the digastric and stylo-hyoid muscles, and lies between them and the stylo-glossus. It is in contact with the root of the lingual artery; but separated, on reaching the side of the tongue, by the intervention of the hyo-glossus. The lingual artery, till reaching the junction of the body, with the horn, of the hyoid bone, is covered by the integuments, platysma myoides, cervical fascia, lingual nerve, and hyo-glossus muscle : it then bends forward and dives into the substance of he tongue.*

The os hyoides being, in this position, nearly as high in the throat as the jaw, the descent of the mylo-hyoid muscle is very trifling. Hence, by removal of the submaxillary gland, a large hollow is left between the side of the tongue and lower jaw; its roof, formed towards the chin, by the mylo-hyoideus; toward the angle of the jaw by the hyoglossus, which last is intersected by the stylo-glossus. Between this cavity and the carotid arteries the ligament of the angle of the jaw intervenes; and, above the hyo-glossus, the lingual branch of the fifth pair of nerves goes to the tongue. In this cavity, inclosed between the bellies of the digastric muscle and the jaw, lies the submaxiliary gland: besides this conglobate glands, with the facial artery and vein and their submental branches, are lodged in it. This artery rises (from the external carotid) a little below the angle of the jaw; and, after giving off the ascending palatine, and tonsillar branches, reaches the submaxillary gland, and lies in a groove formed in its substance. The facial vein, descending along the outer side of the gland,† commonly discharges its blood into the internal jugular, just below the edge of the digastric muscle.

Deep, behind this cavity, almost opposite the root of the alveolar process of the second molar tooth, the tonsil is situated between the pillars of the fauces, in the angle formed by the stylo-glossus and stylo-pharyngeus muscles; and covered by the palato-pharyngeus. The tonsillar artery sometimes rises from the lingual, generally from the labial, where it passes along the insertion of the stylo-glossus: hence this branch is necessarily short. The external carotid, a little above the origin of the labial artery, lies opposite to the tonsil; the internal, somewhat behind the gland. The glosso-pharyngeal nerve, coming out between the external and internal carotids at the origin of the stylo-pharyngeus, is, with that muscle and the stylo-glossus, quite sunk behind the jaw-bone. But the position of these parts is greatly changed on bending back the head; more especially the submaxillary gland and facial vessels, which are now brought forward, in consequence of the diminution of the cavity naturally existing between

* By an incision directed, below the chin, through the integuments, platysma myoides, fascia, anterior belly of the digastric muscle, the mylo-hyoid, and genio-hyoid, the lingual artery will be exposed lying between the genio-glossus and lingualis muscles.

† That side of the gland nearest the ear.

the jaw-bone and mylo-hyoideus. The external carotid, after passing from behind the stylo-hyoid and digastric muscles, and while lying over the internal carotid, gets attached to the parotid gland, and gives off important branches deep within its substance.* The gland itself is sunk in between the ramus of the jaw and mastoid process: it extends to the root of the meatus auditorius externus, nearly as far as the internal carotid and jugular vein; behind the plate of the jaw, adhering to the internal pterygoid muscle; behind the sterno mastoid; below the angle of the jaw; and (anteriorly) folded over the posterior border of the masseter. Two conglobate glands are commonly connected with the parotid; one situated below the angle of the jaw, and covered by the thin dependent lobe of that gland and cervical facia; the other imbedded in the centre of the gland, opposite the point at which the external carotid divides into the temporal and maxillary arteries. The portio dura of the seventh pair of nerves, in its passage from the stylo-mastoid foramen, lies behind the parotid gland but directly enters its substance; and proceeds, for about half an inch, an undivided trunk downward and forward. On its exit from the skull, it is deep-seated, almost in contact with the art. posterior auris.† The styloid process separates it from the internal carotid and jugular vein. Mid-way between the ramus of the jaw and mastoid process, it is nearly opposite the posterior facial vein and external carotid. At this point, imbedded in the substance of the gland, the nerve sends off branches which separately perforate the gland to their various destinations, upward, forward, and downward. The largest branch, inclining upward and forward, covers with numerous twigs, ere it escapes from the gland, the zygoma and transverse facial artery. The most considerable of these twigs runs mid-way between the zygoma and parotid duct.‡ This duct, directly on emerging from the anterior

---

* The posterior auricular, internal maxillary and temporal. The artery is here crossed by the portio dura.

† Sometimes the posterior auricular and occipital arteries arise from one trunk. In that case, the latter vessel is very near the portio dura.

‡ An incision from the root of the mastoid process downward and forward along the anterior margin of the sterno-mastoid muscle, will expose the portio dura at its exit from the stylo-mastoid orifice. The lobe of the ear must be pulled upward and forward in this operation, and the incision must be deep. The nervus superficialis colli, where, entering the inferior angle of the parotid gland, and the posterior auricular artery, will be divided; and the substance of the gland itself implicated.

margin of the gland, comes in contact with the surface of the masseter muscle ; over which it traverses to the buccinator. A line extended from the superior part of the lobe of the ear to mid-way between the root of the nose and angle of the mouth, will mark its course. Reaching the anterior border of the masseter, it turns over the mass of fat which lies between that muscle and the buccinator; contracts as it perforates the latter ; and enters the mouth between the second and third superior molares, just below the margin of the gum. The facial vein crosses the duct near its termination ; the artery runs nearer to the angle of the mouth. The duct is accompanied throughout by twigs of the facial nerve, all small except the branch before noticed. The socia parotidis, resembling in structure and function the parotid gland, lies on the masseter muscle, connected with that gland, the transverse facial artery and portio dura. Its fluid is poured into the parotid duct, which it touches or overlaps. It is situated between the parotid duct and zygoma, nearer to the parotid gland than the middle of the masseter. It varies much in figure and extent. One or two conglobate glands are frequently seen near it. The transverse artery of the face, arising from the external carotid just before its division into the maxillary and temporal, sometimes from the latter, deep in the substance of the parotid gland, almost as low as the origin of the duct, perforates the anterior margin of the gland ; takes its course upward ; and, ere reaching the middle of the masseter, is placed mid-way between the parotid duct and zygoma, and beneath the socia parotidis. Here it divides into numerous branches, distributed either to the ramifications of the portio dura, or to the facial muscles ; and anastomosing with branches of the temporal, internal maxillary, and facial arteries.

Originating from the frontal veins, the anterior facial descends from the root of the nose, touches the insertion of the orbicularis palpebrarum, and lower down is covered by its fibres. From hence, it passes in an oblique course to the anterior border of the masseter; intersecting, about an inch below the internal angle of the eye, but nearer towards the zygoma, the infra-orbitar hole ; and having the levator anguli oris interposed between it and the infra-orbitar nerve and artery : it afterwards crosses, in inclining towards the angle of the jaw, the parotid duct. During its whole course, the vein lies nearer the ear than the artery ; though parallel, and almost in contact with it from the angle of the mouth to the base of the jaw : here both vessels are covered by the platysma myoides. The artery, mounting over the jaw (by the antetior margin of the masseter) upwards and in a serpentine course, gives off midway between the base of the jaw and mouth, the art. labialis surperficialis; then, at a short distance from each other, the inferior and superior coronary arteries, deeply imbedded in the substance of the lips, and separated from the cavity of the mouth only by the lining membrane. The artery now continues to climb along the side of the nose, external to the levator anguli oris, but covered by the levator labii superioris alæque nasi. After supplying the ala nasi, it receives, at the root of the nose, additions from the orbit; and the final branch reaches the forehead to communicate with the ophthalmic and temporal arteries.

The recess between the margin of the orbit and tendon of the orbicularis palpebrarum contains the lachrymal sac, of oblong figure; its tapering extremity downward: itself and the ducts from the puncta lachrymalia covered by the fibres of that muscle. From its most depending extremity originates the nasal duct, which opens into the nostril by a small rounded orifice, half an inch posteriorly to the ascending plate of the superior maxilla, and almost opposite the centre of the inferior spongy bone. The margin of the aperture is loose, membranous, sometimes puckered.*

The internal maxillary artery, rising from the external carotid, deep in the substance of the parotid gland, dives behind the ramus of the jaw, and there gives off its branches.† The external carotid now assumes the name of temporal; becomes more superficial; mounts over the zygomatic process of the temporal bone; and is imbedded in the cellular substance covering the aponeurosis of the temporal muscle.

Hitherto, we have been considering the anatomy of the

---

* In introducing a probe into this orifice, the instrument, properly curved, is to be passed along the floor of the nostril with its concavity towards the antrum, until the point has passed the ascending plate of the maxillary bone: then it is to be rotated until the point looks upward and outward. In making this turn, the probe's point should be kept close to the side of the nostril; and when the turn is made, the handle of the instrument should be depressed, its body and point elevated. Thus the latter will enter the orifice of the nasal duct. If the probe be obstructed, by catching a fold of the nasal membrane, the instrument must be retracted, and moved slightly away from the nostril's side.

† Principally art. meningea media, maxillaris inferior, temporales profundæ, exterior and interior, infra-orbitalis, pterygo-palatina, and nasalis. The last, however, is rather the continuation of the trunk.

head and neck in the *adult:* the structure of the neck in the *young* subject next claims our attention. Mr. Burns allots to it a distinct chapter.

The differences existing between the child and adult in the structure of the neck are considerable. If in a child, aged about one year, the basis cranii be parallel to the horizon, three fingers' breadth exists between the chin and sternum: the hyoid bone is on a level with the base of the jaw, and two fingers' breadth behind the chin: the laryngeal cartilages do not yet project. There is one finger's breadth from the hyoid bone to the base of the cricoid cartilage: half a finger's breadth may be allowed for the thyroid gland: consequently, between this and the sternum, one finger's breadth and a half will remain. If the head be turned back, five fingers may be laid between the chin and sternum; four between that bone and the hyoid; two between the sternum and thyroid gland.* The thymus gland, peculiar to early life, is situated in the anterior mediastinum, directly behind the sternum, interposed between it and the left subclavian vein and arteria innominata. At this age, it mounts about half an inch above the sternum. To the top of this bone, the upper margin of the subclavian vein runs parallel; the lower crosses the origin of the great aortic branches. Between the fourth and half of an inch above the chest, rarely lower, the art. innominata turns to the side of the trachea. Two fingers' breadth above the clavicle, three below the angle of the jaw, the sterno-mastoid and omo-hyoid muscles cross each other. The common carotid lies just behind the point of intersection, and divides one finger's breadth above this point; two below the angle of the jaw, and almost opposite the summit of the thyroid cartilage, into external and internal. Hence, the relation of this division to the layrnx is the same as in the adult; but its distance from the jaw is much greater, from the non-developement of the teeth and alveolar processes. On the same principle, the superior thyroid, lingual, labial, inferior pharyngeal and occipital arteries, arise lower than the digastric muscle from the external carotid; and this vessel is proportionately nearer the facial nerve than in adult age. Three circumstances are principally to be to be noted in contemplating the peculiarities

* By stretching the membrane which connects the hyoid bone and thyroid cartilage, half a finger's breadth is gained in the distance between that bone and the base of the cricoid cartilage.

of structure which distinguish the neck in infancy and mature age: the great distance existing between the carotic bifurcation and angle of the jaw, in the child; the exposure of the primary arterial branches; and the large space between the jaw and the crossing of the omo-hyoid and sterno-mastoid muscles. These peculiarities are explained by the shortness of the ramus of the lower jaw and narrowness of both it and the upper jaw before the alveolar processes are evolved. It is only about the period of permanent dentition, that the jaws deepen, the angle of the lower is carried back, its rami elongate, and the parts begin gradually to assume the characters of adult conformation.

The smallness of the trachea and larynx of the child in proportion to the body is particularly striking. Richerand has found that there exists scarcely any perceptible difference of size in the larynx and glottis of a subject from the third to the twelfth year; that, at the period of puberty, the vocal organ begins rapidly to increase; and that, in less than one year after, the rima glottidis of the male acquires a double size.* From this enlargement of the larynx, on arriving at puberty, it results, that the distance between the lower border of the thyroid gland and the sternum is the same in a child of two years as in an adult: and Mr. Burns "uniformly found in a subject just before the age of puberty, an actual measurement of from one quarter to half an inch more between the sternum and thyroid gland, than in the adult." The trachea of course is proportionally longer in early than in mature age. The situation of the thyroid gland being regulated by that of the cricoid cartilage, if the latter be relatively high in the neck, the distance between the gland and sternum must be greater. But as the larynx expands, the cricoid cartilage, and with it the thyroid gland, is depressed, and the distance between the latter and the chest decreases. Hence, it is clear why, in bending back the adult head, the increase of space is principally between the chin and thyroid gland; while, before the evolution of the larynx, such a position augments chiefly the space between the gland and sternum.

* The increase of the size of the glottis after puberty is not so striking in the female as in the male: with the former, it is only in the proportion of seven to five. An interesting paper on this subject is given by Richerand, in the third volume of the *Memoires de la Société Medicale d'Emulation.*

*Observations on the structure of the neck of the edentulous subject* conclude the anatomical portion of the volume. The toothless person, resembling in some points the young subject, in others, the adult with the head turned back, possesses also a character peculiar to advanced life.

In the child, from causes before mentioned, the large vessels about the throat are much exposed; the parotid gland, from the greater space between the angle of the jaw and anterior margin of the sterno-mastoid muscle, broad but short; and, from the accumulation of adipose substance exteriorly to the muscles, there is a plumpness, afterwards lost on the more regular distribution of the fat.

In the adult, the jaws are broad; their circle wide; the space between the angle and mastoid process diminished; the parotid larger but less broad than in the child; the primary carotic branches and styloid process nearly covered by the lower jaw; all the parts uniformly full.

In the edentulous subject, the distance between the palatine plate of the superior maxilla and chin is much lessened by the loss of teeth and alveolar processes; and, but for the length of the inferior jaw, the characters of infantile conformation would prevail. On closing the mouth, the chin is elevated forward; the angle removed from the mastoid process; the breadth of the parotid gland increased; a hollow formed behind the jaw; the styloid process, and large vessels, and nerves about the throat, exposed. The mylo-hyoid muscle, and digastric in its anterior portion, are also stretched, even in the horizontal position of the basis cranii: hence, the submaxillary gland is brought below the base of the jaw. In this the aged subject resembles the adult with the head turned back. The angle of the jaw being elevated in consequence of the removal of the teeth and alveolar processes, the point of intersection of the sterno-mastoid and omohyoid muscles is, in relation to the angle of the jaw, as low in the edentulous, as in the young subject: and a line, drawn from this point to the angle of the jaw, instead of describing the course of the common, and great part of the external, carotid, as in the adult, turns forward from the vessel, and forms with it an acute angle in the edentulous subject.

The plates which accompany this volume are ten in number: seven illustrating points of surgical pathology; the remainder strictly anatomical. The sixth, exhibiting the distributions of the portio dura, is well drawn; but we are particularly pleased with the tenth, wherein the relations of the rima glottidis are clearly exposed.

In concluding this sketch of the surgical anatomy of the head and neck, we have to lament, that it is, in many points, far from being perfect. The facts, recorded by Mr. Burns, indicate acute observation and a profound knowledge of the subject; but their value is greatly lessened by the defect of plan and arrangement which his work every where displays. In some instances, we have endeavoured to remedy this defect, as far as it could be done consistently with the fidelity and objects of criticism. We had ever to recollect, that the observations of Mr. Burns, not our own, were to be detailed. In order to introduce a precise and philosophic character into such intricate investigations, a widely different course from that pursued by Mr. Burns should, in our opinion, be adopted. The mean distance between all the prominent points and remarkable orifices and depressions of the cranium, face, cervical portion of the spine, scapula, clavicle, sternum, and hyoid bone, and their relations to each other, and to the various parts upon the surface, should be first rigorously determined from accurate measurements of several perfectly formed adult skeletons. Similar plans should next be presented from the osseous system of the child at a given age, and from that of the edentulous subject. Afterwards, the variations operated by changes of aspect and posture of the cranium, at these three periods of life, should be separately sketched. These points established, the head and neck of the recent adult subject should be divided into separate and strongly defined regions, and a detached examination of the contents, and relations of the parts, of each region, from the surface to the bones, should be followed by a general view of all the regions combined. The head and neck of the child and of the aged person should be investigated on a similar principle: and, lastly, all the peculiarities resulting from differences of aspect and position, at every period, should be cautiously observed and distinctly noted. A plan thus solid and substantial is more easily sketched than carried into execution. It would demand the profound acquirements and unconquerable perseverance of a Macartney or a Lawrence, to give to such a work the full and luminous developement of which it is susceptible.

The labours of Mr. Burns are, however, as far as they go, alike correct and valuable; nor can we too warmly recommend his work to all classes of the profession. That the sketch just delivered may assist the student in his investigations of a most important part of the human fabric, and prove serviceable to those more advanced, in recalling the ever-fleeting recollection of anatomical facts, we confidently hope: and most earnestly do we call upon those young men, who are now qualifying themselves in the dissecting-rooms of the metropolis, for the practice of an honourable profession, to make surgical anatomy the end and aim of all their studies. This is a knowledge, without which isolated views of the structure and situation of the various organs will avail but little. This is a knowledge which, amid scenes of peril and dismay, when the ignorant retire in confusion and disgrace, will inspire with intrepidity and self-possesson the enlightened surgeon, and acquire for him the confidence, veneration, and gratitude, of all around him.

Our observations on the surgical part of Mr. Allan Burns's work will appear in our next Number.

